# The human brain intracerebral microvascular system: development and structure

**DOI:** 10.3389/fnana.2012.00038

**Published:** 2012-09-13

**Authors:** Miguel Marín-Padilla

**Affiliations:** Departments of Pathology and Pediatrics, Geisel School of Medicine at DartmouthHanover, NH, USA

**Keywords:** EGLM, endothelial cell filopodium, human brain, intracerebral microvascular system, meningeal inner pial lamella

## Abstract

The capillary from the meningeal inner pial lamella play a crucial role in the development and structural organization of the cerebral cortex extrinsic and intrinsic microvascular compartments. Only pial capillaries are capable of perforating through the cortex external glial limiting membrane (EGLM) to enter into the nervous tissue, although incapable of perforating the membrane to exit the brain. Circulatory dynamics and functional demands determine which capillaries become arterial and which capillaries become venous. The perforation of the cortex EGLM by pial capillaries is a complex process characterized by three fundamental stages: (1) pial capillary contact with the EGLM with fusion of vascular and glial basal laminae at the contact site, (2) endothelial cell filopodium penetration through the fussed laminae with the formation of a funnel between them that accompanies it into the nervous tissue while remaining open to the meningeal interstitium and, (3) penetration of the whole capillary carrying the open funnel with it and establishing an extravascular Virchow-Robin Compartment (V-RC) that maintains the perforating vessel extrinsic (outside) the nervous tissue through its entire length. The V-RC is walled internally by the vascular basal lamina and externally by the basal lamina of joined glial cells endfeet. The VRC outer glial wall appear as an extension of the cortex superficial EGLM. All the perforating vessels within the V-RCs constitute the cerebral cortex extrinsic microvascular compartment. These perforating vessels are the only one capable of responding to inflammatory insults. The V-RC remains open (for life) to the meningeal interstitium permitting the exchanges of fluid and of cells between brain and meninges. The V-RC function as the brain sole drainage (prelymphatic) system in both physiological as well as pathological situations. During cortical development, capillaries emerge from the perforating vessels, by endothelial cells growing sprouts analogous to their angiogenesis, entering into their corresponding V-RCs. These new capillaries to enter into the nervous tissue must perforate through the V-RC outer glial wall, a process analogous to the original perforation of the cortex EGLM by pial capillaries. These emerging capillaries are incapable of reentering the V-RCs and/or perforating vessels. As the new capillary enters into the nervous tissue, it becomes surrounded by glial endfeet and carries a single basal lamina (possibly glial). Capillaries emerging from contiguous perforators establish an anastomotic plexus between them, by mechanisms still poorly understood. The capillaries of this anastomotic plexus constitute the cerebral cortex intrinsic microvascular compartment and together constitute the so-called blood-brain-barrier. The intrinsic capillaries are changing and readapting continuously, by both active angiogenesis and reabsorption, to the gray matter neurons developmental and functional needs. The brain intrinsic capillaries are among the most active microvessels of the human body. Unresolved developmental and functional aspects concerning the cerebral cortex intrinsic capillary plexus need to be further investigated.

## Introduction

The CNS vascularization is an ascending process that accompanies the anatomical and functional maturations of its various territories. It starts at the myelencephalon and ascends sequentially through the metencephalon, mesencephalon, diencephalon, and finally the telencephalon (Klosovskii, 1963; Strong, 1964; Bär and Wolff, 1972; Gamble, 1975; Wollf et al., 1975). Important interrelationships between the meningeal tissue and the perforating vessels have been described in a variety of studies (Mall, 1904; Strong, 1964; Pape and Wigglesworth, 1979; Hauw et al., 1975; Nabeshina et al., 1975; Krahn, 1982; Krisch et al., 1982, 1983; Marín-Padilla, 1985, 1988). This paper describes an additional anatomical and functional concerning the entrance of pial capillaries into the nervous tissue and about the interrelationships between intracerebral microvascularization and the neuronal maturation of the cortex gray matter.

The embryonic development of the human brain vascular system is highly complex and involves the sequential formation of various independent, although interrelated, vascular compartments outside as well as inside the cerebral cortex. These embryonic processes establish intracerebral extrinsic and intrinsic microvascular compartments throughout the cortex gray (where most neurons reside) and white matters. Some developmental, anatomical, histological, and functional aspects of these processes remain poorly understood. To more fully understand how the cortex gray matter intracerebral microvascular compartments are developed, we must correlate them with the stratified and ascending maturation of its neuronal, fibrillar, and glial systems (Marín-Padilla, 2011). A better understanding of how these complex vascular and cellular processes will provide valuable insights into the human cerebral cortex basic cytoarchitectural and functional organizations. Such an understanding may also help in interpreting the evolving neuropathology of epilepsy that results from perinatal brain damage (Marín-Padilla, 1996, 1997, 1999, 2000; Marín-Padilla et al., 2002) as well as that of some neurodegenerative encephalopathies (Marín-Padilla and Kopman, 2011).

Capillary angiogenesis (and reabsorption) is a universal process in the vascularization of any tissue. It has been described in a variety of experimental models, including: transparent chambers (Clark and Clark, 1939), corneal implants (Ausprunk and Folkman, 1977; Ausprunk, 1979) tissue cultures (Sholley et al., 1984; Madri et al., 1983), tumors (Ausprunk and Folkman, 1977; Folkman, 1982) and inflammatory processes (Schoefl, 1963; Ryan and Majno, 1977; Majno and Joris, 1978; Cotran, 1982). Some aspects concerning the cerebral cortex extrinsic and intrinsic microvascular compartments of humans and other mammals remain incompletely studied and understood.

This paper explores the embryology, anatomy, histology, and some functional aspects of the human cerebral cortex microvascular system, emphasizing the sequential and interrelated progression of its extracerebral and intracerebral compartments. Also highlighted are the concomitant stratified and ascending structural and functional interrelationships among the cortex gray matter neuronal, microvascular, and neuroglial systems. The paper emphasizes that the classic Golgi's staining method (Golgi, 1873) historically considered to be an excellent tool to study CNS neurons, can also be a successful tool to study the development and morphology of the mammalian brain microvasculature, and that of its fibrous (white matter), protoplasmic (gray matter), and first lamina astrocytes (Marín-Padilla, 1985, 1995, 2011). Cajal used and modified the rapid Golgi method to study the brain of newborn infants (Cajal, 1899a,b, 1890a,b) and I have also modified it to study the human brain's prenatal development. A detail description of this classic technique, including its unique staining capabilities, contributions, advantages and disadvantages, misunderstandings, and technical advices has appeared in a special chapter (The Golgi Reaction). A Personal Quest in a recently published book (Marín-Padilla, 2011).

The observations presented herein are largely based on rapid Golgi and electron microscopic research studies (Marín-Padilla, 1967, 1971, 1978, 1985, 1987, 1988, 1992, 1995, 2011) as well as from literature descriptions (Andres, 1976a,b; Jones, 1970; Pape and Wigglesworth, 1979; Krahn, 1982; Krisch et al., 1983; Krisch and Buchheim, 1984; Mrzljak et al., 1988).

## The human brain's vascular system

The human and mammalian brain's vascular system have three distinct and interrelate components: the extracerebral or meningeal compartment, and the intracerebral dual extrinsic and intrinsic microvascular compartments (Table 1). Their sequential embryonic development begins in the very young embryo with the establishment of the extracerebral meningeal compartment that covers the surface of the growing brain followed by that of the intracerebral extrinsic (perforating vessels) microvascular compartment, from which the intrinsic microvascular compartment (the blood-brain-barrier) subsequently evolves (Table 1).

**Table 1:** **Human cerebral cortex vascular system components**.

1. Extracerebral meningeal compartment
(a) Dural lamella: main venous sinuses
(b) Arachnoidal lamella: main arteries and veins
(c) Pial lamella: pial capillary anastomotic plexus
2. Intracerebral extrinsic microvascular compartment^a^
(a) Perforating capillaries (eventually arterioles and venules)
(b) Virchow-Robin space, the cortex drainage (prelymphatic) system
3. Intracerebral intrinsic microvascular compartment^a^
Intrinsic capillary anastomotic plexus between contiguous perforators, the cortex blood brain barrier (BBB)

a Both, the extrinsic (directly) and the intrinsic (indirectly) intracerebral microvascular compartments evolve from the Pial Capillary Anastomotic Plexus.

**Figure 1:**
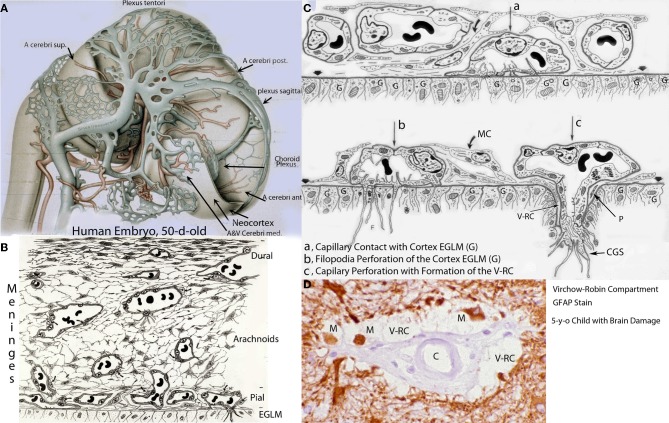
**Various developmental aspects of the human brain embryonic vascular system. (A)** Streeter drawing of a 50-day-old human embryo (Streeter, 1918) showing the meningeal compartment main vessels. The cerebral cortex (neocortex), not yet vascularized, is covered by arachnoidal vessels and its surface appears smooth and without a pial capillary plexus. **(B)** Schematic representation of a 50-day-old human embryo's meninges, showing the vessels of its dural, arachnoidal, and pial lamellae. Also a pial capillary anastomotic plexus covers the cortex surface in close proximity to its external glial limiting membrane (EGLM), composed of glial endfeet covered by basal lamina material. The filopodia of one of the capillaries (lower right on ELGM layer) are perforating and entering into the brain nervous tissue. **(C)** Schematic representation of the three developmental stages of pial capillaries entering into the cortex: (a) pial capillary contact with cortex EGLM with fusion of both vascular and glial basal laminae, (b) endothelial cell filopodia perforation through the fussed basal laminae and entrance into the nervous tissue, and (c) capillary perforation and entrance into the nervous tissue with formation of the extravascular Virchow-Robin Compartment (V-RC) around the vessel. At this stage, some meningeal cells (MC) enter into the V-RC accompanying the vessel (P) and become the source of its smooth muscles. By the continued incorporation of additional glial endfeet around the perforating vessel, the surface EGLM seems to be extending into the brain accompanying it. The V-RC accompanies the perforating vessels through its entire length while remaining open to the meningeal interstitium, functioning as the brain's sole drainage (prelymphatic) system. Perforating vessels remain outside of the nervous tissue and together they represent the brain's extrinsic microvascular compartment. A Glial fibrillary acidic protein-stained brain section of a 5-year-old child with brain damage, showing the V-RC with its central vessel **(D)**, the perivascular spaces (V-RC) with several stained macrophages (M), and its glial stained outer wall. Some macrophages seem to be entering into the V-RC extravascular space. The stained macrophages seem to have phagocytized glial products from the damaged site. Key: G, glial endfeet; V-RC, Virchow-Robin Compartment; M, Macrophages; CGS, perforating capillary growing tip; MC, meningeal cell; P, pericytes.

**Figure 2:**
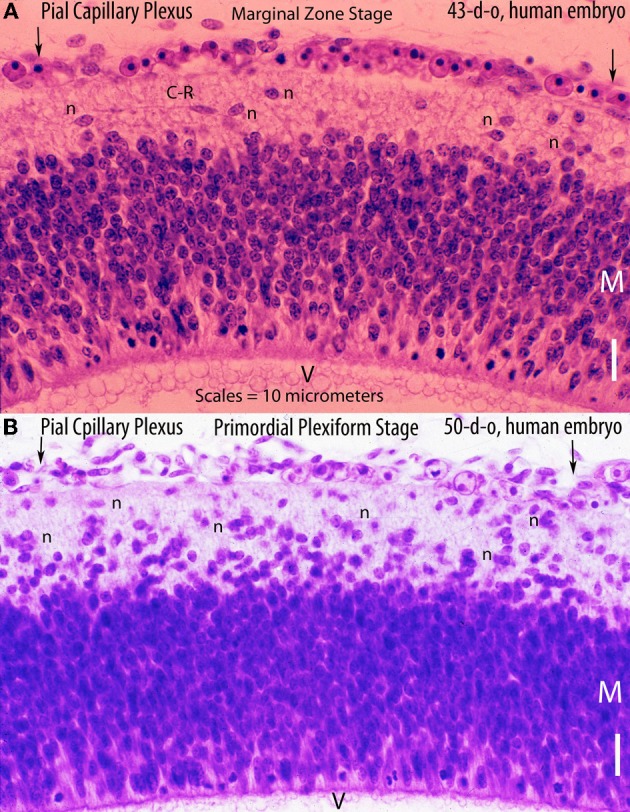
**Hematoxylin and eosin stained sections of the cerebral cortex of (A) a 43-day-old embryo (tubal pregnancy) and of (B) a 50-day-old embryo (hysterectomy) showing the rich pial anastomotic capillary plexus that covers their still-unvascularized cerebral cortex.** The pial capillaries are numerous, very small, and invisible to the naked eye. At this early embryonic age, they still contain nucleated red cells. The embryos cortical developments are at the marginal zone (43-d-o) and the primordial plexiform (50-d-o) stages. A variety of neurons (n) are recognized above the matrix (M) zone, including a horizontal Cajal-Retzius cell (C-R). Key: V, ventricle. Scale = 10 mm.

The extracerebral or meningeal compartment contains three essential constituents: the outer dural, the middle arachnoidal, and the inner pial lamellae (Table 1). The dural lamella contains the main venous sinuses, the arachnoidal lamella contains the brain main arteries and veins, and the pial lamella contains the pial capillary anastomotic plexus (PCAP) (Figures 1A,B and 2A,B). The meningeal compartment, with all its essential vascular components, is already recognizable in 50-day-old human embryos (Streeter, 1918). The establishment of the meningeal compartment occurs before the brain intracerebral microvascularization begins (Marín-Padilla, 1985, 2011).

Most descriptions of the human brain vascular system refer almost exclusively to its meningeal compartment, which has been amply described in the literature. Padget described and beautifully illustrated the complete embryonic development of the human brain dural sinuses and that of its arachnoidal veins and arteries (Padget, 1948, 1957). Although her work remains classic, she makes no mention of the PCAP within the inner meningeal lamella. Moreover, the crucial role that the pial capillaries play in the cerebral cortex intracerebral microvascular development has remained inadequately studied. Using electron microscopic and rapid Golgi preparations, the development, composition, organization, and role the pial capillary plexus plays in establishing the brain intracerebral microvasculature are analyzed and discussed herein.

## The pial capillary anastomotic plexus

**Figure 3:**
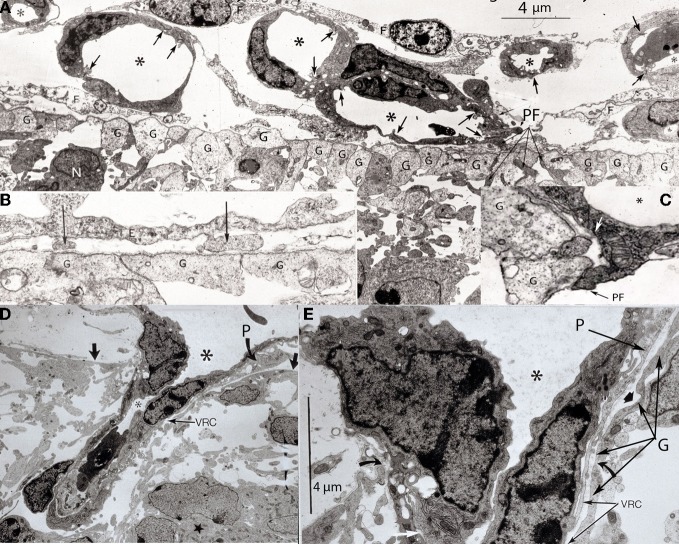
**Various electron photomicrographs, from the brains of 12-day-old hamster embryos, showing various aspects of the perforation of the cerebral cortex external glial limiting membrane by pial capillaries. (A)** Photomicrograph (4 μm-scale) showing that the cerebral cortex delimited by the external glial limiting membrane (EGLM) composed of glial endfeet (G) covered by basal lamina material a several pial capillaries above it. The capillaries (*) are composed of endothelial cells separated by junctions (arrows) and their size varies, including very small (possibly growing) capillaries. One of the capillary has established direct contact with the cortex EGLM, with fusion of both vascular and glial basal laminae, and some endothelial cell filopodia (PF, long fine arrows) that already have perforated through the EGLM and penetrate into the nervous tissues. This pial capillary is a growing one with numerous internal and external filopodia as well as a sliding endothelial cells over its wall. **(B)** Detail of endothelial cell filopodia establishing early contacts with the cortex EGLM. **(C)** Detail of the entrance of an endothelial cell filopodium (PF) into the nervous tissue, through the fused vascular and glial basal laminae, with the formation of a funnel (white arrow) that remains open to the meningeal interstitium. **(D)** Low-power view showing a pial capillary (*) entrance into the nervous tissue through the EGLM (arrows) with the formation of the Virchow-Robin Compartment (V-RC) around it, and the penetration of a meningeal cell (P) into the extravascular space. The growing sprout of the perforating vessels is composed of several advancing endothelial cells. **(E)** High-powered view (4 μm scale) of a pial perforating capillary (*) showing its entrance into the cortex (curved arrows) and the formation of the extravascular V-RC (V-RC) around it. The continuous incorporation of additional glial endfeet (G) to the V-RC outer wall seems as an extension the surface EGLM that keep the vessels extrinsic (outside) of the nervous tissue. The entrance of a pericytes (P) from the meningeal space into the extravascular V-RC is also shown. The V-RC remains open to the meningeal interstitium and function as the brain's sole drainage (prelymphatic) system.

The PCAP is an essential component of the inner meningeal lamellae and hence is extracerebral. Its capillaries represent the source of all the perforating vessels that will enter into the cerebral cortex during both its prenatal and postnatal developments. The pial perforating capillaries will progressively establish the brain intracerebral microvasculature. Eventually, the perforating vessels will participate in the formation of the brain's intracerebral intrinsic microvascular compartment (Table 1). The PCAP is composed of innumerable and closely linked capillaries of variable sizes that cover the entire surface of the developing brain (Figures 1A–C, 2A,B, 3A). The capillary size ranges from 3 to 7 μm in diameter. The PCAP thickness ranges between 7 and 9 μm and is extremely fragile. In addition to the capillary plexus, the inner meningeal lamellae has scattered meningeal cells, including fibroblasts, pericytes, cells of neural crest origin, collagen fibers and interstitial spaces bathed by cerebrospinal fluid (Figures 1C, 3A).

Despite the fundamental role of the PCAP in the intracerebral microvascularization of the mammalian brain, it has not been adequately described in most embryology textbooks (Padget, 1948, 1957; Hamilton et al., 1972; Larsen, 1997; O'Rahilly and Müller, 1999). Why has this important meningeal vascular component been essentially ignored? Perhaps, two facts could explain the oversight. The PCAP capillaries are invisible to the naked eye (Figures 1B,C, 2A,B, 3A). And the meninges removal (customarily done in brain inspection and dissection) invariably carries the entire pial capillary plexus with it, leaving the brain surface smooth and without any apparent vascularization. A strong magnifying glass or a dissecting microscope is necessary to visualize the innumerable small openings throughout its entire surface. These orifices are formed by either entering arterial or by exiting venous vessels. In fact, the openings are the apertures of the Virchow-Robin compartments (V-RCs) that contain the entering and exiting vessels severed by the meninges removal. In the newborn brain, each exiting venous vessel, which has a slightly larger opening than that of entering vessel, is surrounded by 6–8 smaller entering arterial vessels, perhaps defining vascular territories. It is important to emphasize that the intervascular distance among these vascular orifices remains essentially unchanged throughout the entire cortical surface and ranges between 400 and 600 μm. The intervascular distance between these orifices also defines the dimension (width) of the intrinsic microvascular compartment formed among them throughout the cortex gray matter. This intervascular distance, despite the increasing number of perforators and the brain progressive increase in both size and volume, remains essentially unchanged during both its prenatal and postnatal developments (Marín-Padilla, 2011).

The growth and expansion of the PCAP parallels that of the cerebral cortex, and is carried out by the continued incorporation of new capillaries either from the arachnoidal vessels and/or by local active angiogenesis. The size (diameter) of pial capillaries varies, including very small, probably representing growing ones (Figure 3A). There are also growing (sprouting) capillaries characterized by the formation of internal as well as external filopodia, the presence of an occasional mitoses and the incorporation a new endothelial cells into their wall (Figure 3A). These growing capillaries are the one that first establish contact with the cortex EGLM (Figure 3A). Among the capillaries, there are a variety of meningeal cells, collagen fibers, and meningeal interstitial spaces with circulating cerebro-spinal fluid (Figures 1B,C, 3A). Some of these meningeal cells will accompany the perforating vessel into the brain and possibly become the source of its smooth muscles (Figures 1C, 3C,D).

The mammalian cerebral cortex's four early developmental stages (neuroectodermal, marginal zone, primordial plexiform, and pyramidal cell plate early formation) evolve within a still avascular cortex covered by a rich PCAP (Marín-Padilla, 2011). Undoubtedly, the close proximity of pial capillaries to the neuronal and fibrillar elements of these early developmental stages diffuses the necessary oxygen for their survival (Figures 1C, 2A,B, 3A). In humans, a PCAP covering the cerebral cortex is already recognized in 6- and 7-week-old embryos (Figures 2A,B). Their cortical developments are at the marginal zone (6-w-g) and the primordial plexiform (7-w-g) stages, respectively. At this early embryonic age, the pial capillaries still contain numerous nucleated red cells (Figures 2A,B). In the developing human cerebral cortex, and that of other mammals, these early functional stages precede the formation of the pyramidal cell plate that represents a mammalian innovation (Marín-Padilla, 1971, 1978, 1992, 1998). During the subsequent prenatal and postnatal development, and for as long as the cortex is functionally expanding, the PCAP continues to grow covering the expanding cortical surface, as additional pial capillaries as well as additional perforating vessels are incorporated as needed.

## Pial capillary perforation of the cerebral cortex

The PCAP capillaries are surrounded by vascular basal lamina and they are separated from the underlying nervous tissue by the brain external glial limiting membrane (EGLM) that is also entirely covered by basal lamina material manufactured by the glial cells (Marín-Padilla, 1985, 1987, 1988, 2011). Consequently, during the mammalian brain vascularization a remarkable developmental event occurs repeatedly, namely: two different types of tissues covered by their respective and specific basal laminae establish structural and functional interrelationships. Blood capillaries, of mesodermal origin, covered by their own basal lamina contact and penetrate into the brain, which is a neuroectodermal tissue also covered by its own glial basal lamina. How this remarkable dual (contact and subsequent incorporation) event occurs has been seldom described. How these two different -vascular and glial- basal laminae behave during the brain microvascularization is analyzed and described herein.

The developing mammalian cerebral cortex (and CNS) is covered by an EGLM composed of radial glial endfeet, united by gap junctions, which are covered by basal lamina material manufactured by the glia cells (Figures 1B,C, 3A,B). It represents an anatomical boundary that delimits the cerebral cortex (and entire CNS) from surrounding tissues maintaining its anatomical integrity. It represents the original basal plate, covered by basal lamina, of the embryonic neuroectoderm before its closure. Its subsequent closure into a neural tube explains the EGLM superficial (external) location throughout the brain (and CNS). During early embryonic development, the radial glial cells supply the necessary endfeet for the EGLM maintenance and integrity. Later in prenatal and during postnatal cortical maturation, the glial endfeet necessary for its progressive expansion, maintenance, and integrity are supplied by first lamina special glial (astrocytes) cells (Marín-Padilla, 1995). Any EGLM injury, which often occurs in perinatal hypoxic and/or isquemic brain damage, invariably results in the formation of pathological lesions (leptomeningeal heterotopias) as neuronal (neurons and fibers) elements escape into the meningeal interstitium through the resulting ruptures (Marín-Padilla, 1996).

Although the human cerebral cortex begins its neuronal development around the 6th week of gestation, its intracerebral microvascularization does not begin until the 8th week. The mammalian cerebral cortex primordial (embryonic) neuronal and fibrillar organizations resemble that that of amphibians and reptiles (Marín-Padilla, 1971, 1978). The pyramidal cell plate, which is a mammalian innovation, becomes incorporated into this primordial cortical organization separating its elements into upper (first lamina) and lower (subplate) zones. In the developing human cerebral cortex, the pyramidal cell plate begins its incorporation into the developing gray matter, around the 8th week of gestation and is nearly completed by the 15th week (Marín-Padilla, 2011). All pyramidal neurons precursors, attracted by the protein Reelin, must arrive at the first lamina and establish functional contacts with Cajal-Retzius neurons (Marín-Padilla, 1992). All true pyramidal neurons will remain functionally anchored to the first lamina for life. Consequently, they must elongate their apical dendrite, first anatomically, to accommodate the arrival of subsequent pyramidal neurons and later functionally, by adding functionally active synaptic membrane, during the neuron ascending functional maturation (Marín-Padilla, 1992, 2011). The cerebral cortex intracerebral microvascularization starts through proximal (ventral) cortical regions and progresses through the distant (dorsal) regions, following and paralleling the arrival and penetration (under the EGLM) of neurons and fibers from extracortical sources (Zecevic et al., 1999; Marín-Padilla, 2011) as well as the subsequent arrival and incorporation of pyramidal neurons into its developing gray matter.

A rich pial anastomotic capillary plexus covering the entire cerebral cortex is already recognized in 6- and 7-week-old human embryos, which is fully established before the initiation of the cortex intracerebral microvascularization (Figures 2A,B). To enter into the cerebral cortex, the pial capillaries must perforate through the EGLM. Pial capillaries are the only vessels capable of perforating through the cortex EGLM to enter into the nervous tissue. However, they are incapable of perforating the membrane to exit the brain. During development, circulatory dynamics determine which capillaries become entering arterial and which capillaries become exiting venous. Throughout both prenatal and postnatal cortical development, and for as long as the brain is functionally expanding, new pial perforating vessels continue to enter into the cortex by the same mechanism; circulatory dynamics and increased functional demands will continue to determine their arterial or venous function as well as their eventual size and diameter. In other CNS regions, nerves can also perforate the EGLM to enter as well as to exit the nervous tissue (Andres, 1976a,b).

The pial capillary perforation of the cortex EGLM is a complex and sequential developmental process. It consists of three distinct and sequential events: (1) pial capillary contact with the EGLM with fusion of vascular and glial basal laminae, (2) endothelial cell filopodia perforation of the EGLM through the fused laminae, and (3) whole capillary perforation of the EGLM with penetration into brain and the formation of the extravascular Virchow-Robin compartment (V-RC).

Some pial capillaries approach and establish directly contact the cortex EGLM, followed by the fusion of vascular and glial basal laminae at the contact site (Figures 1C, 3A–C). Pial capillaries endothelial cell filopodia are the first to establish contact with the EGLM (Figures 3B,C). The fusion of both basal laminae is followed by the endothelial cell filopodium perforation through the EGLM and its entrance into the nervous tissue (Figures 1C, 3A,C). The individual filopodium perforates through the fused basal laminae, thus preserving the brain's anatomical integrity. The fused laminae create a funnel around the perforating filopodium that will accompany it into the brain while remaining open to the meningeal interstitial space (Figures 3A,E).

Subsequently, the entire pial capillary penetrates into the nervous tissue through an opening that is surrounded by the fussed basal laminae, retaining the brain anatomical integrity (Figures 1C, 3D,E). The original funnel formed between the fused basal laminae accompanies the perforating capillary into the brain (Figures 1C, 3D,E). The extravascular space that surrounds the entering vessels will maintain it extrinsic (outside) of the nervous tissue while remaining open to the meningeal interstitium (Figures 3D,E). This extravascular space, known as the V-RC, is recognized in both developing and adult brains (Figures 1C,D, 3D,E). The V-RC is externally walled by the glial basal lamina and internally by the vascular basal lamina (Figures 1C,D, 3D,E). The incorporation of the additional glial endfeet covered by basal lamina, to the growing V-RC, appears as if the superficial EGLM has extended into the cortex around the perforating vessel (Figures 1C, 3D,E). The incorporation of additional glial endfeet around the perforating vessel, maintains it extrinsic to the nervous tissue throughout its entire length (Figures 1C, 3D,E).

Some measurements (size and diameter) of the pial perforating vessels have been carried in the motor cortex of newborns infants, using rapid Golgi preparations (Marín-Padilla, 1985, 1988, 2011). In Golgi preparations, the vessel outer endothelial wall is covered by silver precipitate, hence rendering it visible. Therefore, the measurements represent the vessel outer diameter. In the newborn motor cortex, the diameter of perforating capillaries (the most common and abundant type) ranges between 5 and 8 μm; that the arterioles from 10 to 15 μm; and that venules from 16 to 20 μm. The size variability reflects their different functional demands. It must be emphasized that because only pial capillaries are capable of perforating the cortex EGLM to enter into the brain, all perforating vessels (arterioles, venules, arteries, and veins) subsequently recognized throughout the cerebral cortex prenatal and postnatal developments have evolved from original pial capillaries (Marín-Padilla, 2011). Circulatory dynamics and functional demands will determine the perforating vessels eventual size or diameter. During prenatal development, the eventual balance between perforating arterial and venous vessels is constantly changing and they adapt to the growing brain functional needs. During postnatal brain maturation, additional functional demands will continue to influence the original perforating vessels sizes and diameters.

## Intracerebral extrinsic microvascular compartment

**Figure 4:**
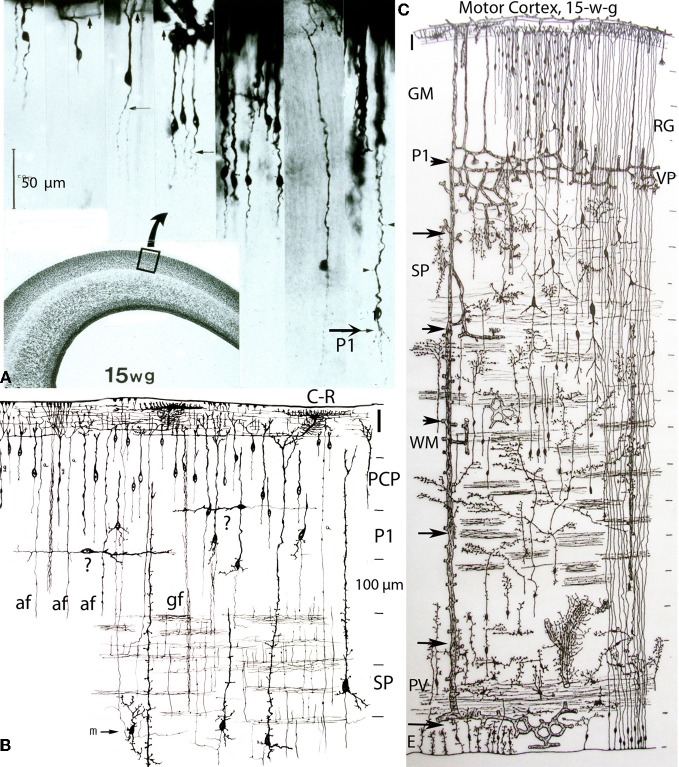
**Composite of a photomicrograph and two camera lucida drawings, from rapid Golgi preparations, illustrating various aspects of the developing cerebral cortex (motor region) of a 15-week-old human embryo.** At this age, the thickness of the cerebral cortex, at the motor region, is around 2 mm. **(A)** Montage of photomicrographs (50 μm scale) showing, at least 7 different pyramidal cell strata with neurons raging in size from 40 μm for the upper, smaller, and last to reach the Cajal-Retzius cells of the first lamina, to 275 μm for the lower, larger, and first to reach the Cajal-Retzius cells. At this age, the deepest and older pyramidal neurons (P1) have started to develop short basal dendrites (arrow) and a few apical dendritic spines (small arrow heads) indicating the beginning of their ascending functional maturation. The pyramidal neurons of the upper strata are still immature with smooth and spineless apical dendrites and descending axon that start to reach the white matter. Cajal-Retzius horizontal axonic fibers (small arrows) are also recognized within the first lamina. The formation of the pyramidal cell plate that started around the 8th week of gestation is nearly complete at this age. **(B)** Composite of camera lucida drawings (100 μm scale) from rapid Golgi preparations comparing the size, location, interrelations, and organization of the neuronal, and fibrillar elements of the first lamina (I), the pyramidal cell plate (PCP) and the subplate zone (SP). Only the deepest and older pyramidal neurons have started their ascending functional maturation (P1) by developing basal dendrites and their descending axons have entered the white matter. At this age, horizontally migrating neurons (labeled “?”) are first recognized through the cortex lower pyramidal cell strata; we now know these migrating cells are the precursors of the cortex inhibitory neurons. These neurons become incorporated into the deepest, older and maturing pyramidal cell (P1) stratum and will be its future inhibitory (basket, bi-tufted, and chandelier cells) neurons. Also at this age, the subplate zone (SP) deep primordial neurons (pyramidal-like and Martinotti cells) start to lose their original attachment to the Cajal-Retzius cells of the first lamina. **(C)** Montage of camera lucida drawings (100 μm scale) reproducing the entire thickness of the human motor cortex (at this gestational age), illustrating the size, morphology, distribution, and organization of its basic neuronal, fibrillar, microvascular, and glial elements. Intrinsic capillary anastomotic plexuses, between adjacent perforators, are recognized through the ependymal (E), paraventricular (PV), white matter (WM), subplate (SP), and lower gray matter (GM) zones (arrows). Some perforating vessels reach the paraventricular zone, few reach the white matter and many more reach the gray matter. Fibrous (white matter) and early protoplasmic (gray matter) astrocytes are recognized around their capillaries. At this age, the cortex gray matter starts to develop its first intrinsic microvascularization (VP) through its deepest, older and maturing pyramidal cell (P1) stratum. The remaining gray matter (GM) pyramidal cell strata are still immature at this age. Also, at this age, the deep primordial neurons of the subplate zone start to lose their original functional attachment to first lamina Cajal-Retzius cells. The white matter is crossed by bundles of corticipetal and corticofugal axonic fibers and by numerous ascending glial and neuronal precursors. Glial cell precursors of fibrous astrocytes and oligodendrocytes are recognized accompanying the white matter fibers in both directions. Also illustrated are radial glial cells (RG) attached to the ependyma, with ascending filaments that reach the cortex surface with endfeet incorporated into the EGLM as well as small glial cells precursors still attached to ependyma.

**Figure 5:**
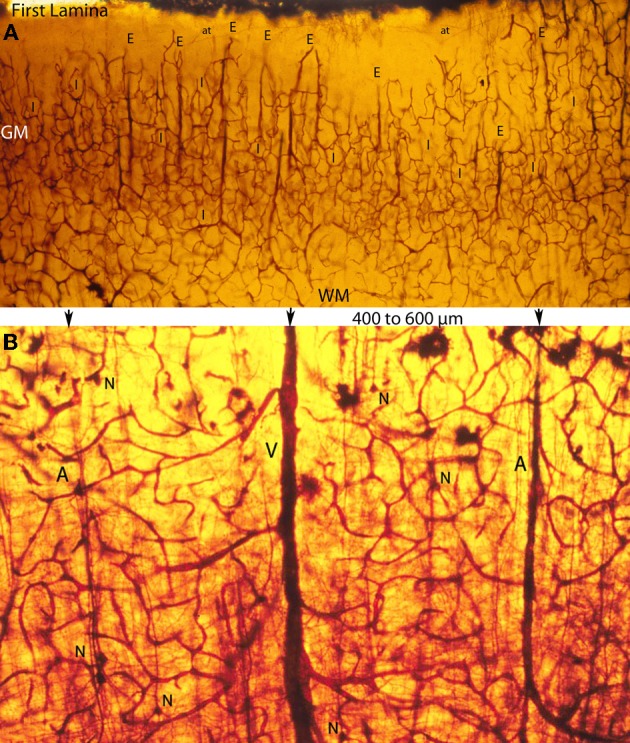
**Photomicrographs of rapid Golgi preparations from the motor cortex of newborn infants, illustrating lower (A) and higher (B) power views of the intracerebral extrinsic (E) and intrinsic (I) microvascular compartments as well as their close structural and functional interrelationships. (A)** Photomicrograph of the newborn motor cortex gray (GM) and white (WM) matter intracerebral extrinsic and intrinsic microvascular compartments, illustrating the abundance of capillaries, their three-dimensional organization, as well as the nearly constant intervascular distance between the extrinsic perforating vessels (E) and the similar dimension of the intrinsic (I) microvascular compartments established between them. Both essentially similar measurements are believed to represent physiological constants needed for the normal neuronal functional activity of the mammalian brain. The white matter (WM) compared with the gray matter (GM) has fewer capillaries and larger intercapillary spaces. A few horizontal axonic fibers (at) from Cajal-Retzius are recognized running through the first lamina. **(B)** High-powered view of the gray matter extrinsic and intrinsic microvascular compartments between a central venule (V) and two adjacent arterioles (A), separated by 400–600 μm. This intervascular distance also delineates the dimension of the intrinsic microvascular compartment between the perforating vessels. The intrinsic microvascular compartment is an anastomotic capillary plexus with small intercapillary spaces where neuron (N) resides; its capillaries have a single basal lamina and together they represent the blood brain barrier. These capillaries may be incapable of responding to brain inflammatory insults and/or participate in inflammatory processes (See also Figure 7A).

**Figure 6:**
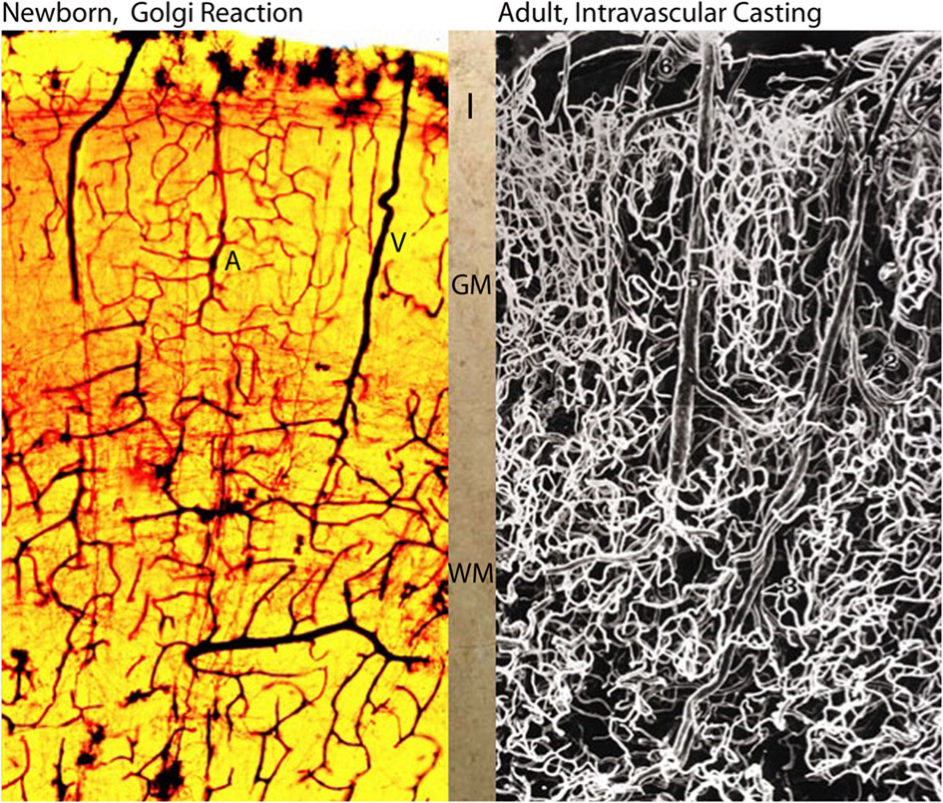
**Photomicrographs comparing the intracerebral extrinsic and intrinsic microvascular compartments of a newborn (A) and adult (B) human brains.** The reproduction in **(A)** is from a rapid Golgi preparation of the motor cortex of a newborn infant and that in **(B)** is from an intravascular casting of an adult human brain, from the work of Duvernoy et al. (1981). Despite the significant differences in brain size weight (newborn ca. 410 g and adult ca. 1,350 g) the overall dimension, vascular composition and structural organization of their intracerebral extrinsic and intrinsic microvascular compartments are remarkable similar. These structural and organizational similarities mirror the similar developmental and physiological constrains that endure through the prenatal and postnatal functional maturations of cortical neurons Marín-Padilla (2011). In both brains **(A, B)**, there are more intrinsic capillaries with smaller intercapillary spaces in the gray matter than in the white matter. The abundant of intrinsic capillaries through the cortex gray matter protect the functional activity of its neurons, in both normal and abnormal conditions. Key **(A)**: I, first lamina; GM, gray matter; WM, white matter; A and V, arterial and venous vessels; and in **(B)** 6, pial vein; 5, venule; 1, arteriole; 3, deep arteriole; 2, recurrent arteriole.

**Figure 7:**
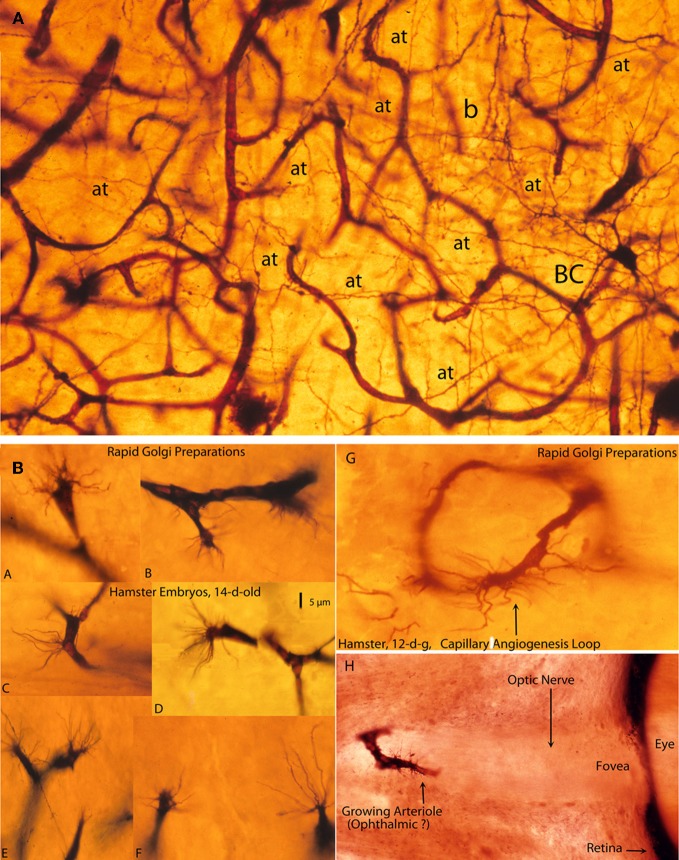
**Photomicrographs from rapid Golgi preparations of a newborn infant (A) motor cortex and (B) from the brain of 12- and 14-day-old hamster embryos, (A) a high-powered, three-dimensional view of the complex and rich intrinsic capillary anastomotic plexus of a newborn motor cortex showing the abundance of capillaries, their small intercapillary spaces and a stellate basket cell (BC) in one of them.** This basket cells has numerous horizontal, oblique, and vertical axonic terminals (at) and a recognizable (b) pericellular basket. Together, these intrinsic capillaries represent the brain's blood-brain barrier and, physiologically, they are the responsible for the normal functional activity of cortical neurons; and, **(B)** Golgi preparations, from 12- and 14-day-old hamster embryos brains showing various examples (A–F) of growing capillaries (angiogenesis) showing the polyp-like ending sprouts of advancing endothelial cells with numerous terminal searching filopodia. Also illustrated are: a growing capillary loop (G) between adjacent vessels and a growing vessel (H) in the center of the unstained optic nerve of the eye of a 12-day-old hamster embryo, with terminal searching filopodia. Also illustrated are the eye's fovea and pigmented retina.

All perforating vessels within V-RCs, throughout the cerebral cortex, constitute the brain's intracerebral extrinsic microvascular compartment. The thick rapid Golgi preparations (150–200 μm) are excellent for visualizing the unique three-dimensional organization of cortex extrinsic microvascular compartment (Figures 4C, 5A,B, 6A,B). The vertical perforating vessels are clearly distinguishable from the intrinsic anastomotic capillary plexus established between them (Figures 5A,B, 6A,B). Similar images of the intracerebral microvascular system are obtained with the intravascular injection of different color dyes (Pape and Wigglesworth, 1979). The intervascular distance between entering and exiting vessels remains relatively constant throughout both prenatal and postnatal cortical development (Figures 4C, 5A,B, 6A,B, 7A). The intervascular separation among perforators also determines the overall dimension (width) of the intrinsic capillary plexus established among them.

The extravascular V-RC accompanies the perforating vessels throughout their entire length while remaining open to the meningeal interstitium. As the cortex thickness increases, the length of both the perforating vessels and the V-RCs also increase accordingly (Figure 4C). The perforating vessels within the V-RCs are the only vessels capable of responding to inflammatory insults within the brain. Inflammatory cells will exit the perforating vessel into the V-RC and, to enter into the nervous tissue, they must perforate through its outer glial wall. Inflammatory cells are also capable of returning to the V-RCs reversing this process. However, they cannot reenter into the perforating vessels. Consequently, their only possible outlet (drainage) from the brain tissue is through the V-RC's open communication with the meningeal interstitium.

The V-RC's open communication with the meningeal interstitium permits the interchange of fluids and cells between brain and meninges, quite noticeable during inflammatory processes. The fluid and cellular interchange between brain and meninges has been described in several studies (Casley-Smith et al., 1976; Krisch and Buchheim, 1984; Pile-Spelman et al., 1984). A substance injected into the brain will be eventually recognized in the neck lymphatic nodes. A possible direction for the injected substance, usually carried by macrophages, could be: brain tissue, V-RC, meningeal interstitium, perivascular lymphatics of meningeal vessels, lymphatic system and, eventually, the neck lymphatic nodes.

Moreover, because the mammalian brain (and CNS) lacks a lymphatic system, the V-RCs are the brain's only drainage system under both normal and abnormal conditions (Marín-Padilla, 2011; Marín-Padilla and Kopman, 2011). Consequently, the V-RCs function as the brain prelymphatic drainage system. Invariably, in brain injuries and/or diseases, inflammatory cells (mostly macrophages) fill the local V-RCs, including those that are moving toward as well as returning from the brain-injured site. In both directions, they must go through the V-RC outer glial wall. Perhaps, the relatively short and constant distance between the V-RCs (400–600 μm), determines the length inflammatory cells must travel to and from the injured site as well as the time required (Figures 5A,B). Despite the immense number of perforating vessels throughout the cortex, the V-RC's drainage capacity as a prelymphatic system is precarious, and may become inefficient if the brain damage is either extensive and/or recurrent (Marín-Padilla and Kopman, 2011). Moreover, because the V-RCs lacks valves, the only way inflammatory cells can move toward their meningeal opening is through muscular contractions (palpitations) of the central vessels, an obviously inefficient mechanism, as well as by their own power. Because some macrophages may not survive the distance between contiguous V-RCs, they could die in situ and undergo progressive enzymatic degradation. Such residual necrotic proteinaceous debris can elicit additional and recurrent inflammatory responses, perpetuating an unresolved situation. Perhaps, this process could explain the evolving nature of the neuropathological alterations of some degenerative encephalopathies (post-traumatic and Alzheimer encephalopathies) and, also, account for the increasing number of lesion (Marín-Padilla and Kopman, 2011).

It is extraordinary that the intervascular distances among perforating vessels as well as the width of the intrinsic capillary plexuses formed between them should remain essentially similar throughout the human brain entire development, despite its remarkable increase in both size and volume (Figures 6A,B). Considering that an adult human brain weighs about three times than that of a newborn infant, these vascular similarities are remarkable. Perhaps, they might be expected since the development of the intracerebral microvascular system, its functional requirements, and overall structural organization remain essentially unchanged through the human brain prenatal and postnatal development. Considering that each exiting venous vessel is surrounded by, at least, eight entering arterial ones and that their overall separation remain unchanged, the total number perforating vessels throughout the cerebral cortex should run into the thousands. Since the surface of the newborn cortex is about 7000 mm^2^ (Blinkov and Glezer, 1968), the number of perforating vessels should be around 63,000 per brain. In the adult human cerebral cortex, with a surface area of 16,350 mm^2^ (Blinkov and Glezer, 1968), the number of perforating blood vessels should be around 147,000. Although, these numbers are mere approximations, they provide an idea of the richness and remarkable activity of the cerebral cortex extrinsic and intrinsic microvascular compartments.

By the end of the 8th week of gestation, perforating vessels and corresponding V-RCs are recognized through the entire human cerebral cortex. At this embryonic age, the brain is still small, weighing less than 2 g and therefore, there are very few perforating vessels. In the course of cortical development, the number of perforators and V-RCs will progressively and exponentially increase throughout the growing brain.

The 15th week of gestation represents an important landmark for the human cerebral cortex development and functional maturation (Figure 4). At this age, the ascending migration of pyramidal neurons precursors, from ependymal to first lamina, is nearly complete (Figure 4A). All pyramidal neurons within the gray matter have their terminal dendritic bouquets functionally anchored to Cajal-Retzius cells within the first lamina (Marín-Padilla, 1992). The deepest, older, and larger pyramidal neurons were the first to reach the cortex first lamina and are the first to start their ascending functional maturation that begins at this gestational age (Figures 4A,C). At this age, the superficial and smaller pyramidal neurons are still immature (spineless) and will be the last to mature functionally (Figure 4A). Their ascending and sequential functional maturation will progress from lower to upper levels (Marín-Padilla, 2011).

At this embryonic age, only the deepest pyramidal neurons have developed basal dendrites and a few apical dendritic spines, indicating their starting functional maturation (Figure 4A). However, their apical dendrite upper segment remains undifferentiated, smooth, and spineless. The beginning of the ascending functional maturation of the gray matter first pyramidal cell stratum coincides with the establishment of the first intrinsic capillary plexus of the gray matter, between the local contiguous perforating vessels (Figures 4A–C). The descending axons of these deep maturing pyramidal neurons have reached the underlying white matter and are, probably, starting to reach subcortical centers and establish functional contacts. At this age, the descending axons of the remaining gray matter upper pyramidal neurons are barely reaching the white matter.

Another significant event that occurs at this embryonic age is the presence of horizontally migrating cells through the gray matter lowers pyramidal cells strata (Figure 4B). Today, these horizontally migrating cells, which have arrived from extracortical sources, are considered to be the precursors of the cortex inhibitory neurons. At this age, these neurons, although still undifferentiated, become incorporated into the deepest maturing pyramidal cell stratum (Marín-Padilla, 1969, 1970, 2011). During subsequent gestational ages, they become to be essentially recognized as basket, bi-tufted, and/or chandelier inhibitory neurons (Marín-Padilla, 1990, 2011). It is also important to emphasize that, at this gestational age, the primordial large neurons of the subplate zone (pyramidal-like and Martinotti cells) are also starting to lose their functional attachment to the first lamina (Figures 4B,C). As they become disconnected from the first lamina, these subplate neurons are progressively transformed into deep interstitial interneurons with a function, still essentially unknown. In my opinion, at this age the human cerebral cortex starts to function as a mammalian brain (Marín-Padilla, 2011).

At the 15th week of gestation, intrinsic anastomotic capillary plexus between contiguous perforating vessels are also recognized throughout the paraventricular, white matter, and subplate zones as well as through the gray matter lower region (Figure 4C, arrows). Glial endfeet processes, from fibrous as well as protoplasmic astrocytes, already surround the intrinsic capillaries of these cortical regions, suggesting functional activity (Figure 4C). Others cortical elements are also recognized at this age. The ependymal layer has numerous radial glia cells with ascending filaments that reach the cortex surface terminating in endfeet that are incorporated into the EGLM as well as numerous shorter glial cell precursors still attached to the ependymal layer (Figure 4C). The paraventricular (matrix) zone has large venous vessels surrounded by numerous glial endfeet as well as numerous free-ascending glial cell precursors. A single bundle of horizontal fibers, of still unknown origin and function, is also recognized through this zone (Figure 4C). The white matter zone is crossed by several bundles of horizontal corticipetal and corticofugal axonic fibers and by numerous ascending glial cell precursors that appear to follow the axonic fibers in both directions. They could represent the precursors of white matter fibrous astrocytes as well as oligodendrocytes (Figure 4C).

There are more perforators reaching the gray matter (where most neurons reside) than those reaching the white matter (Marín-Padilla, 1999, 2011). In the newborn infant brain, the number of perforators reaching the gray matter is roughly 6–8 for each one that reaches the white matter (Figures 4C, 5A, 6A,B). As gray matter neurons increase their functional activity, the number of perforators reaching them increases significantly, maintaining a constant intervascular distance among them as well as the ratio (1–8) between venous and arterial perforators. During the cortex postnatal maturation, this ratio will further increase, reflecting the cortex's gray matter increasing functional activity and size. The gray matter's intrinsic microvascular compartment, comparing with that of the white matter, has more capillaries and smaller intercapillary spaces (Figures 5A,B, 6A,B, 7A). In general, the gray matter's richer intrinsic microvasculature and its direct and shorter connections to the pial capillary plexus protect its neurons from perinatal hypoxia and/or ischemic brain damage. Especially, in cases when the underlying white matter has been extensively damaged (Marín-Padilla, 1967, 1999, 2000, 2011; Marín-Padilla et al., 2002). Often, the surviving neurons of the affected gray matter become abnormal because they have lost many arriving corticipetal fibers and their axons have been both destroyed within the damaged white matter (Marín-Padilla, 1997, 1999). In infants born prematurely, the white matter is often more damaged than the gray matter, for similar reasons. The advantages of the gray matter richer intrinsic microvasculature may also protect the adult cerebral cortex neurons from a variety of injuries.

The possible reasons for the synchronous developments of the cortex intrinsic capillary plexus and the starting of pyramidal neurons functional maturation throughout the developing gray matter remain unknown. This concomitant process will be repeated through the subsequent ascending functional maturation of the gray matter pyramidal neurons and possibly during the brain postnatal maturation (Marín-Padilla, 2011). My impression is that the starting functional maturation of the deep pyramidal neurons (appearance of basal dendrites and of dendritic spines) by the arrival of corticipetal (thalamic) fibers determines the formation of the local intrinsic capillary plexus providing for the physiological (functional) demands of local neurons. Guidance molecules as well as growth factors should also play a significant role in these developmental coincidences.

## Intracerebral intrinsic microvascular compartment

During the cerebral cortex functional maturation, capillaries emerge from perforating vessels, puncture through the V-RC outer glial wall, enter directly into the nervous tissue, and establish an intrinsic anastomotic capillary plexus between contiguous ones (Figures 5B, 6A,B, 7A). The paraventricular zone is the first to start its intrinsic microvascularization. This is followed by the progressive, sequential, and ascending intrinsic microvascularization of the developing white matter and the subplate zone. The gray matter is the last cortical region to start its ascending intrinsic microvascularization that coincides with the ascending maturation of its deepest and older pyramidal neurons.

Capillaries emerging from the perforating vessels, to enter into the nervous tissue, must first puncture through both the vascular (perforating vessel) and the V-RC outer glial wall basal lamina. As the capillary enters into the nervous tissue, it will carry only a single basal lamina. The intrinsic capillary single basal lamina could be glial in nature, since glial cells endfeet immediately and completely surrounds it. The intimate interrelationships between the intrinsic capillary endothelial cells wall and the surrounding glial cell processes undoubtedly play important roles in maintaining and controlling the neurons physiological demands and functions. This glial envelope may also explain the unique permeability features of cortex intrinsic capillary (blood brain barrier). In the human cerebral cortex, the first intrinsic capillary anastomotic plexus of the gray matter starts to develop at the 15th week of gestation (Figure 4C) surrounding its deep maturing neurons (Figures 5B, 7A). The fundamental functional role of the brain intrinsic capillaries is to provide all the energy needs to the maturing gray matter neurons. Together, they represent the so-called blood brain barrier (BBB). In my opinion, the gray matter intrinsic capillaries do not respond to inflammatory insults and/or participate in the inflammatory processes.

During cortical development, these capillaries are continuously growing and remodeling the intrinsic plexus and responding to the neurons functional needs. They grow by active capillary angiogenesis and are remodeled by capillary reabsorption (Figure 7B). Growing brain capillaries are characterized by the presence of endothelial cell sprouts with numerous searching filopodia that resemble polyps (Figure 7B). Although this type of capillary grow has been known for some time (Klosovskii, 1963), it has received little attention (Marín-Padilla, 1988). The tip of growing capillary is composed of two or three sliding (advancing) endothelial cells with numerous terminal filopodia that seem to be evaluating the surroundings for clues to determine the vessel direction and final destination (Figure 7B). Growing intrinsic capillaries are capable of establishing new as well as eliminating old anastomotic connections as well as establishing loops and/or bridges between adjoining vessels and/or between capillaries at different cortical levels (Figure 7B). The result is the establishment of tri-dimensional intrinsic capillary plexus among the gray matter neurons (Figure 7A). The neurons occupy the intercapillary spaces maintaining intimate anatomical and functional interrelationships with the capillaries (Figure 7A). In Golgi preparations, a thin and single process, of variable length, attached to the capillary wall characterizes a reabsorbing capillary (Marín-Padilla, 2011). Rapid Golgi preparations illustrate beautifully the unique tri-dimensional features of the human cortex gray matter intrinsic capillary plexus, where most neurons reside (Figure 7B). Similar type of capillary growth has been recognized in other organs outside the cerebral cortex, such the developing eye (Figure 7B).

During the brain's prenatal and postnatal development and functional maturation, the number of new perforating vessels as well as that of intrinsic capillaries increased exponentially. Throughout the developing gray matter, the width of the intrinsic capillary plexus (ranges between 400 and 600 μm) became determine by the intervascular distance among contiguous perforators. The capillaries of the intrinsic anastomotic plexus undergo continuous remodeling by both active capillary angiogenesis and reabsorption as they adjust to the brain functional needs. Within their corresponding compartments, the growth and the number of these microvessels follows and parallels the extraordinary anatomical and functional expansion of the human brain. Although the cortex gray matter intrinsic microvascular compartment is continuously changing and readapting to the neurons' functional needs, there are no significant local differences between them in the infant and the adult human cerebral cortex (Figures 6A,B).

Capillaries emerge from the perforating vessels by endothelial cell growing sprouts by a process analogous to their angiogenesis (Figure 7B). Original capillaries emerging from the perforating vessels are able of entering into the brain by puncturing through the V-RC outer glial wall but they seem incapable of reentering into it. The capillary perforation of the V-RC outer glial wall could be a process analogous to the original perforation of the cortex EGLM by pial capillaries (Figures 1C, 3A,E). Actually, the V-RC outer glial wall represents an extension of the brain's superficial EGLM (Figures 1C, 3D,E). Because capillaries from contiguous perforators have already perforated through their corresponding V-RC outer glial walls to enter into the nervous, there is no need for them to reenter into the V-RCs. Circulatory dynamics will determine which of these intrinsic capillaries become arterial and which ones become venous.

How the brain's intrinsic capillaries become interconnected with one other and reestablish a common lumen remains poorly understood. We have been unable of resolving this complex event using only the rapid Golgi method. A possible alternative will be to use electron-microscopic montages of these interconnections, although it will require the examination of hundreds (perhaps thousands) of sequential preparations, a nearly impossible task. Perhaps, the use of novel electron microscopic techniques may be of great help in clarifying some of these complex developmental events (DeFelipe, 2010).

**Figure 8:**
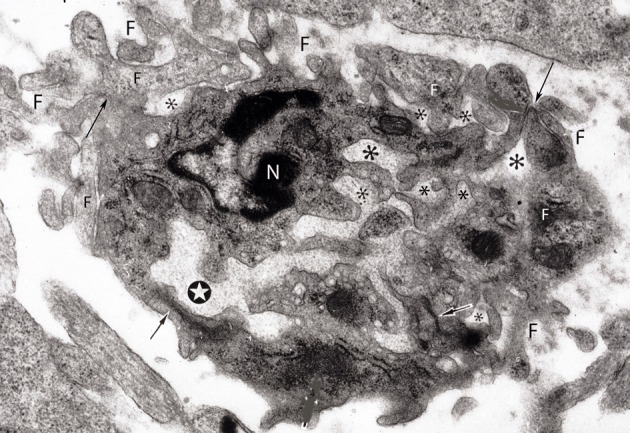
**Electron photomicrograph of a small (3 × 4 μm) and complex capillary from the cerebral cortex of a 12-day-old hamster embryo showing a main vessel surrounded by several attached and non-attached filopodia (F).** The vessel has a main lumen (white star) and several non-communicating additional smaller lumens (*) filled with basal lamina-like material. At least four tight junctions (arrows) are recognized among the endothelial cells. Two of the tight junctions are between the main vessel endothelial cells and the other two junctions are between endothelial cells that appear to be outside the vessel. One endothelial cell of the main vessel has a recognizable nucleus (N). This complex vessel is interpreted as representing the confluence of advancing filopodia and lamelopodia from approaching capillaries that are in the process of establishing intercommunications between them. In the ensuing capillary, the additional lumina will coalesce with the main lumina establishing a common one and the peripheral filopodia will be incorporated into the new capillary wall.

In electron microscopic studies of 12- and 14-day-old hamster brains (Marín-Padilla, 1988), I have observed small (3 × 4 μm) and capillaries that appear to be composed of the incorporation of several endothelial cell terminals (Figure 8). They have a small central lumen and several additional smaller ones unconnected to the main one. Both the main and accessory lumina are filled with basal lamina-like material. These complex capillaries are also surrounded by endothelial cell filopodia, covered by basal laminae, some attached to the main vessel, while others are unattached. These complex capillaries could represent the confluence of advancing endothelial cells' filopodia from approaching one. Advancing endothelial cell filopodia, from approaching capillaries, could be the first elements to establish contacts that, eventually, could result in the formation of a common lumen between them.

## Conclusions

The observations presented in this paper concerning the human cerebral cortex microvascularization are preliminary. Additional studies will be necessary to reconfirm the findings presented herein and to incorporate additional new ones. The developing brain intrinsic capillaries, through their ongoing angiogenesis, reabsorption, and remodeling are probably among the most active microvessels of the human body. Since the brain's intrinsic microvasculature plays a transcendental role in all human activities, it is regrettable that some of its embryological, anatomical, histological and functional aspects should remain incompletely understood.

The development of an anastomotic capillary plexus between contiguous perforators implies a series of events, not yet clearly understood. Here are few of the questions that, in my opinion, need to be further explored and elucidate: are the brain intrinsic capillaries capable, after exiting a VRC, of re-entering into another one? How do these capillaries perforate through the V-RCs outer glial wall to enter into the nervous tissue? Are these capillaries capable of exiting the perforating vessel but incapable reentering another one? What is the nature of the single basal lamina that surrounds the brain intrinsic capillaries? How do these intrinsic capillaries encounter each other within the nervous tissue, become interconnected and establish a common lumen between them? What mechanisms control these vascular processes? How are intrinsic capillaries reabsorbed and/or eliminated? What mechanisms regulate the intrinsic capillary' adaptation to the neurons' functional needs? What are the renewal capabilities of brain intrinsic capillaries throughout the individual life? All these questions need to be further explored.

Some final comments concerning the similar intervascular distance between contiguous perforators and hence, the similar width of the intrinsic microvascular compartments through the cortex's gray matter. It is remarkable that both the intervascular distance between perforator and, hence, the width of the intrinsic capillary plexus should remain unchanged throughout the brain prenatal and postnatal maturations. Specially, considering the brain progressive and significant increase in both size and volume. In my opinion, both the intervascular distance among and the width of its intrinsic capillary plexus represent a physiological constant needed for the brain physiological (functional) needs. The amount of oxygen required by the gray matter neurons functional activity may become determine by the width of the intrinsic capillary plexus where neurons reside. The distance a macrophage could travel between contiguous V-RCs may also determine their similar separation. Otherwise macrophages will perish without reaching their destination and disintegrate in situ (Marín-Padilla and Kopman, 2011). Macrophages must reach the V-RCs and exit the brain though their meningeal opening. Macrophages can neither re-enter the perforating vessels. An interesting physiological possibility will be that the constant intervascular distance between perforators and the width of the cortex intrinsic microvascular compartment predicts the eventual width (roughly 500 μm) of some of the functional vertical columns of Mountcastle found throughout the cortex (Mountcastle, 1978). Perhaps, a combination of all these possibilities plays a significant role in the brain physiological and functional activities. Additional studies are needed to elucidate the possible nature of this proposed physiological constant. Finally, it should be emphasized that the constant intervascular distance between contiguous perforators found throughout the brain gray matter (where most neurons reside) is not applicable to the white matter because a fewer number of perforators reach it. And, also, because the intrinsic capillary plexus throughout the white matter has fewer vessels and their intercapillary spaces are also much larger (Marín-Padilla, 2011).

After the submission of the present article, an excellent work has been published in Science Translational Medicine (“A Paravascular Pathway Facilitates CSF Flow Through the Brain Parenchyma and the Clearance of Interstitial Solutes, Including Amyloid b”, Jeffrey J. Iliff, et al. Sci. Transl. Med. 4: 1-11, 2012) that, with newer technologies, has corroborated that the extravascular (and extracerebral) compartment established around the cerebral cortex extrinsic vessels do function as the brain drainage (prelymphatic) system.

### Conflict of interest statement

The author declares that the research was conducted in the absence of any commercial or financial relationships that could be construed as a potential conflict of interest.
